# TNFAIP3 ameliorates the degeneration of inflammatory human nucleus pulposus cells by inhibiting mTOR signaling and promoting autophagy

**DOI:** 10.18632/aging.104160

**Published:** 2020-11-20

**Authors:** Jie Chen, Yufei Ma, Zhijie Yang, Haiyang Lan, Guangliang Liu, Ye Zhang, Huiqiang Xia, Xiaofang Wang, Fei Han, Xiaolin Tu, Bo Liu

**Affiliations:** 1 Department of Orthopedic Surgery, The First Affiliated Hospital of Chongqing Medical University, Chongqing 400016, China; 2 Laboratory of Skeletal Development and Regeneration, Institute of Life Sciences, Chongqing Medical University, Chongqing 400016, China

**Keywords:** TNFAIP3, human nucleus pulposus cells, inflammation, extracellular matrix, mTOR signaling

## Abstract

Autophagy is involved in degenerative diseases such as osteoarthritis and disc degeneration. Although, tumor necrosis factor α-induced protein 3 (TNFAIP3) is well-known as a key regulator of inflammation and autophagy, it is still not clear whether TNFAIP3 regulates autophagy to protect from human disc cells degeneration. We hypothesize that TNFAIP3 may also regulate autophagy to inhibit pro-inflammatory cytokines expression in human nucleus pulposus cells (NPCs). In this study, TNFAIP3 expression was increased in degenerative disc tissue as well as LPS-stimulated human NPCs, and the effect of TNFAIP3 in LPS-induced NPCs was further explored. The results demonstrated that pro-inflammatory cytokines expression in TNFAIP3-His cells was decreased, while it was increased in TNFAIP3-siRNA cells. Further molecular mechanism research showed that TNFAIP3-siRNA cells enhanced the phosphorylation of mammalian target of rapamycin (mTOR) and inhibited autophagy. Meanwhile, after treatment of TNFAIP3-siRNA cells with the mTOR inhibitor Torin1, the level of autophagy increased and the decrease of extracellular matrix was reversed. In summary, overexpressed TNFAIP3 can promote autophagy and reduce inflammation in LPS-induced human NPCs. Moreover, autophagy triggered by TNFAIP3 can ameliorate the degeneration of inflammatory human NPCs, providing a potential and an attractive therapeutic strategy for degenerative disease.

## INTRODUCTION

As a prominent health problem, inter-vertebral disc degeneration (IVDD) is the main cause of lower back pain (LBP) that severely affects the daily work and life quality [1–2]. Many risk factors, including nutrition, smoking, mechanical loading, aging, genetic factors and others may induce IVDD [3–7]. However, the inducing causes and molecular mechanisms of IVDD remain largely unknown. Excessive inflammation and autophagy in nucleus pulposus cells (NPCs) may play vital roles in the process of IVDD [8]. It has been reported that various pro-inflammatory cytokines, such as IL-1β, IL-6 and TNF-α induce the degradation of aggrecan and collagen, leading to structural instability of the spine and inducing autophagy in the annulus cells as well [9]. The introduction of autophagy promotes extracellular matrix synthesis and further alleviates intervertebral disc degeneration caused by the excessive inflammatory response [10]. The above researches suggest a potential and an attractive therapeutic strategy against IVDD, which targets the inhibition of the inflammatory response and/or promotion of extracellular matrix production by increasing autophagy of intervertebral disc cells.

Autophagy occurs during tissue and organ formation, but also has a critical role in the progress of degenerative diseases, such as osteoarthritis, Alzheimer's disease and IVDD [11–13]. Either higher or lower level of autophagy has a controversial function reported in degenerative intervertebral disc cells [13, 14]. Additionally, some studies showed that inflammatory cytokines promote autophagy in degenerative disc cells [9]. However, the precise role of autophagy in inflammatory human NPCs has not been reported.

Tumor necrosis factor α-induced protein 3 (TNFAIP3) is a negative regulatory factor to prevent inflammation [15] and plays a critical role in the anti-inflammatory activation of certain cells [16]. Simultaneously, TNFAIP3 also decreased the secretion of inflammatory corpuscles by promoting autophagy in Ankylosing spondylitis [17]. So far, the relationship between TNFAIP3, inflammation and autophagy signaling in human NPCs is not clear. Therefore, we investigated whether TNFAIP3 regulates inflammatory cytokines production and extracellular matrix degradation in human NPCs, and the underlying mechanism of this protein to regulate the autophagy signaling. Surprisingly, we found that TNFAIP3 ameliorates inflammatory responses and extracellular matrix degradation by inhibiting the mTOR signaling and promoting autophagy in human NPCs.

## RESULTS

### Upregulation of TNFAIP3 expression in human nucleus pulposus tissues

**Figure 1:**
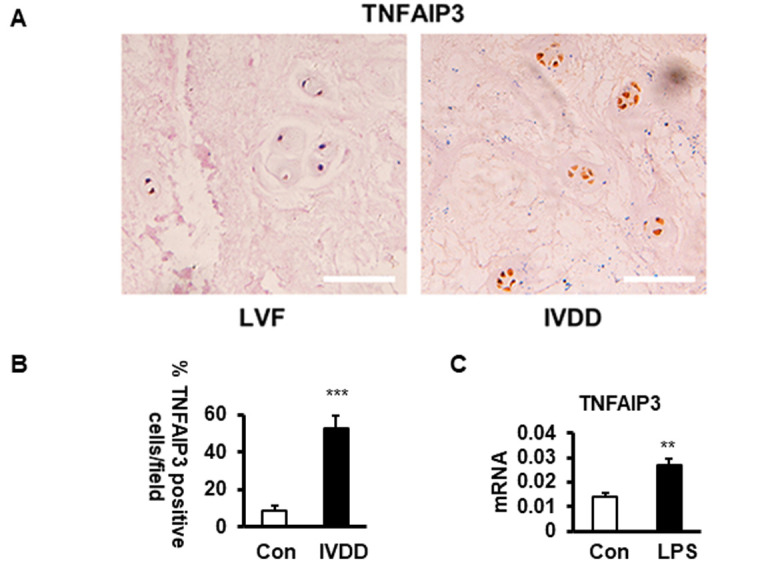
**The expression of TNFAIP3 in human nucleus pulposus tissues.** (**A**, **B**) TNFAIP3 expression was measured by immunohistochemistry from the IVD section from LVF and IVDD patients (magnification × 100, n>5). (**B**) Human NPCs expressing TNFAIP3 were enumerated from the above sections, results are expressed as a percent of the total number of NPCs. (**C**) TNFAIP3 expression was measured at the mRNA level by qPCR from the primary cell culture of the NPCs from LVF patients. Con and LPS, cell culture representatively treated with no and with LPS, data are shown by the mean ± standard deviation from 3 independent replicates. *** P<0.01, ** P<0.05, * P>0.05 by t-test. TNFAIP3, tumor necrosis factor α-induction protein 3; IVD, intervertebral disc; LVF, patients with lumbar vertebral fracture as non-IVDD controls; IVDD, patients with intervertebral disc degeneration; LPS, lipopolysaccharide; Con, normal human NPCs culture from LVF patients with no LPS treatment; LPS, the normal human NP cell culture treated with LPS at 200 ng/mL for 24 h.

To detect whether TNFAIP3 is expressed in IVDD, we tested the TNFAIP3 expression level in human NPCs with different grades of degeneration in IVDD patients. The control group was obtained from patients of lumbar vertebral fracture (LVF) without IVDD. The results of immunohistochemistry revealed that the expression of TNFAIP3 protein in human NPCs of patients with IVDD was significantly increased compared with the control counterparts (Figure 1A, 1B). LPS treatment mimicked the introduction of IVDD as in our previously established LPS-induced IVDD cell model (Aging 2019, 11:7294), which also increased the expression of TNFAIP3 with IVDD introduction (Figure 1C). The results demonstrated that TNFAIP3 expression is upregulated in IVDD patients and LPS-stimulated NP cells, suggesting that TNFAIP3 may be involved in the process of IVDD.

### TNFAIP3 reduces pro-inflammatory cytokines expression in LPS-stimulated human NPCs

**Figure 2:**
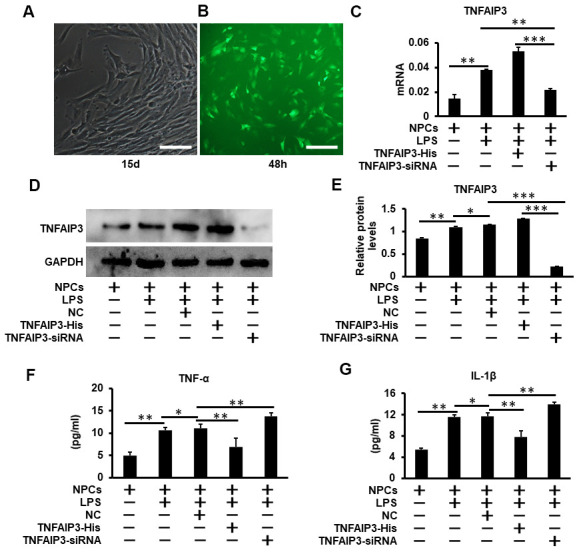
**TNFAIP3 reduces pro-inflammatory cytokines expression in LPS-stimulated human NPCs.** (**A**) Human primary nucleus pulposus cells isolated from patients with lumbar fractures (LVF), amplification × 100. (**B**) Human nucleus pulposus cells were transfected with Ad-TNFAIP3 for 48 h. (**C**–**E**) TNFAIP3 responses to inflammatory stimulation. After treatment with LPS for 24 h, the mRNA and protein expression levels of TNFAIP3 were measured by RT-qPCR and Western blot, respectively. (**F**, **G**) After transfected with TNFAIP3-His and TNFAIP3-siRNA for 24 h, then stimulated with LPS for 24 h, the expression levels of TNF-α, IL-1β were tested by ELISA. Data are represented by the mean ± standard deviation of 3 independent experiments. *** P<0.01, ** P<0.05, * P>0.05 by the Student t-test. Error bars indicate standard deviations. NC, negative control (adenovirus vector without TNFAIP3); TNFAIP3-His, Adenovirus with TNFAIP3 overexpression; TNFAIP3-siRNA, Adenovirus with TNFAIP3 knockdown. TNFAIP3-His +LPS, human NPCs transfected with TNFAIP3-His and then treated with LPS. TNFAIP3-siRNA +LPS, human NPCs transfected with TNFAIP3-siRNA and then treated with LPS.

As a key molecule in the negative regulation of inflammatory response, TNFAIP3 limits the expression of inflammatory cytokines such as TNF-α, IL-1β [15]. As shown in (Figure 2A), primary human NPCs isolated from patients of lumbar vertebral fracture (LVF) were mainly polygonal, irregular fusiform, and the 2^nd^ passaged cells were used for the experiment. The results of fluorescence microscopy showed that the transfection efficiency reached 80% after 48 hours of transfection of NPCs with Ad-TNFAIP3 (Figure 2B). To further confirm the effect of TNFAIP3 on human NPCs, the mRNA and protein levels of TNFAIP3 in LPS-stimulated human NPCs were detected. The expression of TNFAIP3 was higher in LPS-stimulated NPCs compared with non-treated cells. TNFAIP3 siRNA decreased TNFAIP3 levels compared with LPS-stimulated NPCs (Figure 2C–2E). However, there was no difference in the expression of TNFAIP3 between the LPS group and the LPS transfected with the adenovirus vector without TNFAIP3 group, indicating that the adenovirus vector does not affect cell function. We then detected whether the knocking down of TNFAIP3 expression affects the expression of pro-inflammatory cytokines TNF-α and IL-1β. ELISA assay of the supernatants of these cell cultures displayed that LPS increased the expression of TNF-α and IL-1β, but was reduced back to a normal level with overexpressing TNFAIP3 in the human NPCs. In contrast, both TNF-α and IL-1β were significantly up-regulated in TNFAIP3 knockdown NPCs (Figure 2F, 2G). Thus, these results indicated that TNFAIP3 decreased the expression of pro-inflammatory cytokines (TNF-α, IL-1β) in LPS-stimulated human NPCs.

### TNFAIP3 promotes autophagy in LPS-stimulated human NPCs

**Figure 3:**
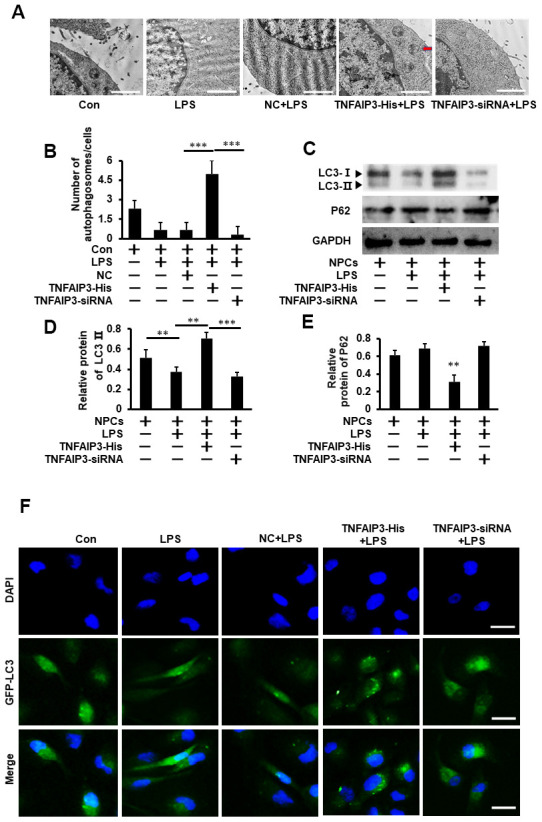
**TNFAIP3 promotes autophagy in LPS-stimulated human NPCs.** (**A**) The effect of TNFAIP3 on autophagy in LPS-stimulated human NPCs. Morphological observation of autophagosome under transmission electron microscope, amplification × 15,000, n>3. (**B**) The number of autophagosomes. (**C–E**) The NPCs were the primary cell cultures from LVF patients without IVDD. Con, no LPS treatment; LPS+TNFAIP3-His and LPS+ TNFAIP3-siRNA, TNFAIP3 and its siRNA were delivered into the NPCs by adenovirus expressing TNFAIP3-His and TNFAIP3-siRNA, respectively. Western blot analysis of LC3II and P62 expression in human NPCs (**F**) Human NPCs were transfected with adenovirus containing GFP-LC3 and the formation and distribution of GFP-LC3 punctate were observed under confocal microscopy, amplification × 800. Data are represented by the mean ± standard deviation of 3 independent experiments. *** P<0.01, ** P<0.05, * P>0.05 by the Student’s t-test.

Cell autophagy is involved in the pathogenesis of degenerative diseases, including osteoarthritis and IVDD [13]. To determine whether TNFAIP3 regulates autophagy in inflammatory human NPCs, the autophagosomes was observed by the transmission electron microscopy (TEM). The number of autophagosomes in TNFAIP3-His+ LPS treatment group were more than that in TNFAIP3-siRNA + LPS or LPS treatment group (As shown in red arrow in Figure 3A). Western blot results showed that the protein expression of LC3 II was significantly increased and P62 expression was decreased in TNFAIP3-His + LPS compared with TNFAIP3-siRNA + LPS or LPS treatment group (Figure 3C–3E), indicating the activation of autophagy in human NPCs. Furthermore, the LC3 puncta calculation experiments were performed by Fluorescence-based detection of LC3 isoforms in human NPCs. The results showed that the LC3 puncta were significantly increased in the TNFAIP3-His and TNFAIP3-His + Torin1 treatment group (Figures 3F, 5G). These results demonstrated that TNFAIP3 may induce autophagy in the LPS-stimulated human NPCs.

### TNFAIP3 inhibits mTOR signaling in the inflammatory human NPCs

**Figure 4:**
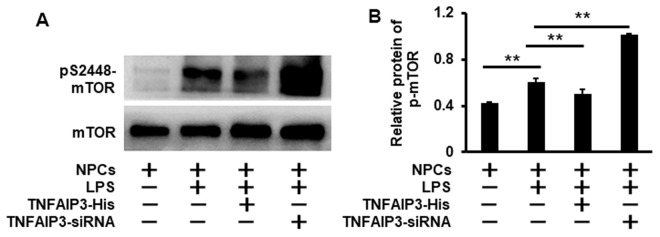
**Knockdown of TNFAIP3 increases mTOR signaling in LPS-treated human NPCs.** (**A**) The mTOR signaling was measured by Western blot in LPS-stimulated human NPCs at the phosphorylation level of mTOR at S2448. (**B**) Relative phosphorylation level of the mTOR. Data are represented by the mean ± standard deviation of 3 independent replicates. *** P<0.01, ** P<0.05, * P>0.05 by the Student’s t-test.

As mTOR is a major negative regulator of autophagy, we hypothesized that TNFAIP3 could inhibit mTOR signaling for the introduction of autophagy. The mTOR phosphorylation at S2448 in the inflammatory NPCs was detected by Western blot. As expected, LPS increased the phosphorylated mTOR level. In contrast, phosphorylated mTOR at S2448 significantly increased in TNFAIP3 knockdown cells and decreased in TNFAIP3 overexpression cells, suggesting TNFAIP3 specifically inhibits mTOR signaling (Figure 4A, 4B). Thus, we infer that TNFAIP3 may decrease phosphorylation level of mTOR after LPS stimulation in human NPCs.

### TNFAIP3 promotes autophagy by inhibiting mTOR signaling in inflammatory human NPCs

**Figure 5:**
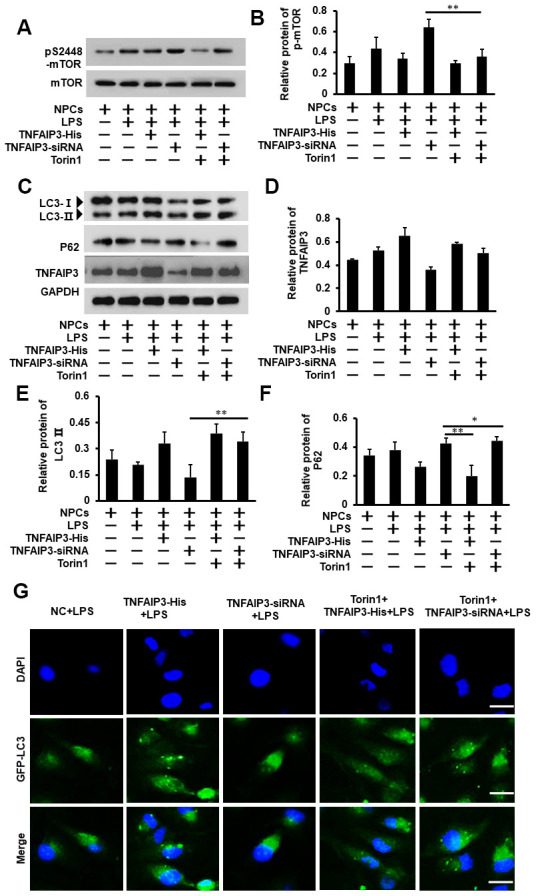
**Inhibition of mTOR signaling ameliorates autophagy in the inflammatory human NPCs.** (**A**, **B**) Torin1 decreases mTOR signaling in inflammatory human NPCs. The phosphorylation level of mTOR at S2448 was measured by Western blot (**A**). Torin1 treated cells at 20 nM for 24 h. (**B**) With its quantification normalized to mTOR level. (**C**, **D**) Inhibition of mTOR signaling increased autophagy by Western blot for LC3II protein level. (**D**) With its quantification normalized to GAPDH for TNFAIP3. (**E**, **F**) The relative quantification of LC3II and P62 protein. (**G**) Human NPCs were transfected with adenovirus containing GFP-LC3 and the formation and distribution of GFP-LC3 punctate were observed under confocal microscopy, amplification × 800. Data are represented by the mean ± standard deviation of 3 independent replicates. *** P<0.01, ** P<0.05, * P>0.05 by the Student t-test.

To understand the molecular mechanism of TNFAIP3 on autophagy induction in inflammatory human NPCs. Torin1, a selective ATP-competitive mTOR inhibitor that can strongly induce autophagy (J Biol Chem 2009; 284:8023-32) was used to evaluate the effect of TNFAIP3 on both mTOR signaling and autophagy in IVDD. Again, LPS induced inflammatory response with higher expression of pS2448-mTOR, which was further enhanced by TNFAIP3 knockdown. However, Torin1 treatment at 20 nM for 1 day shutdown both the increase to non-inflammatory levels (Figure 5A, 5B). Thus, TNFAIP3 inhibits mTOR signaling in this cell model. After Torin1 treatment, western blot results showed that Torin1 reversed the level of LC3 protein in TNFAIP3-siRNA cells + LPS (Figure 5C, 5E), but the protein expression of P62 was not significant in Torin1 treatment (Figure 5C, 5F), indicating the activation of autophagy in human NPCs. In addition, the LC3 puncta were increased in the Torin1 treatment group compared with TNFAIP3-siRNA + LPS group (Figure 5G). The above results suggested that TNFAIP3 induces autophagy through inhibiting mTOR signaling.

### TNFAIP3 inhibits inflammatory response and promotes extracellular matrix expression in human NPCs

**Figure 6:**
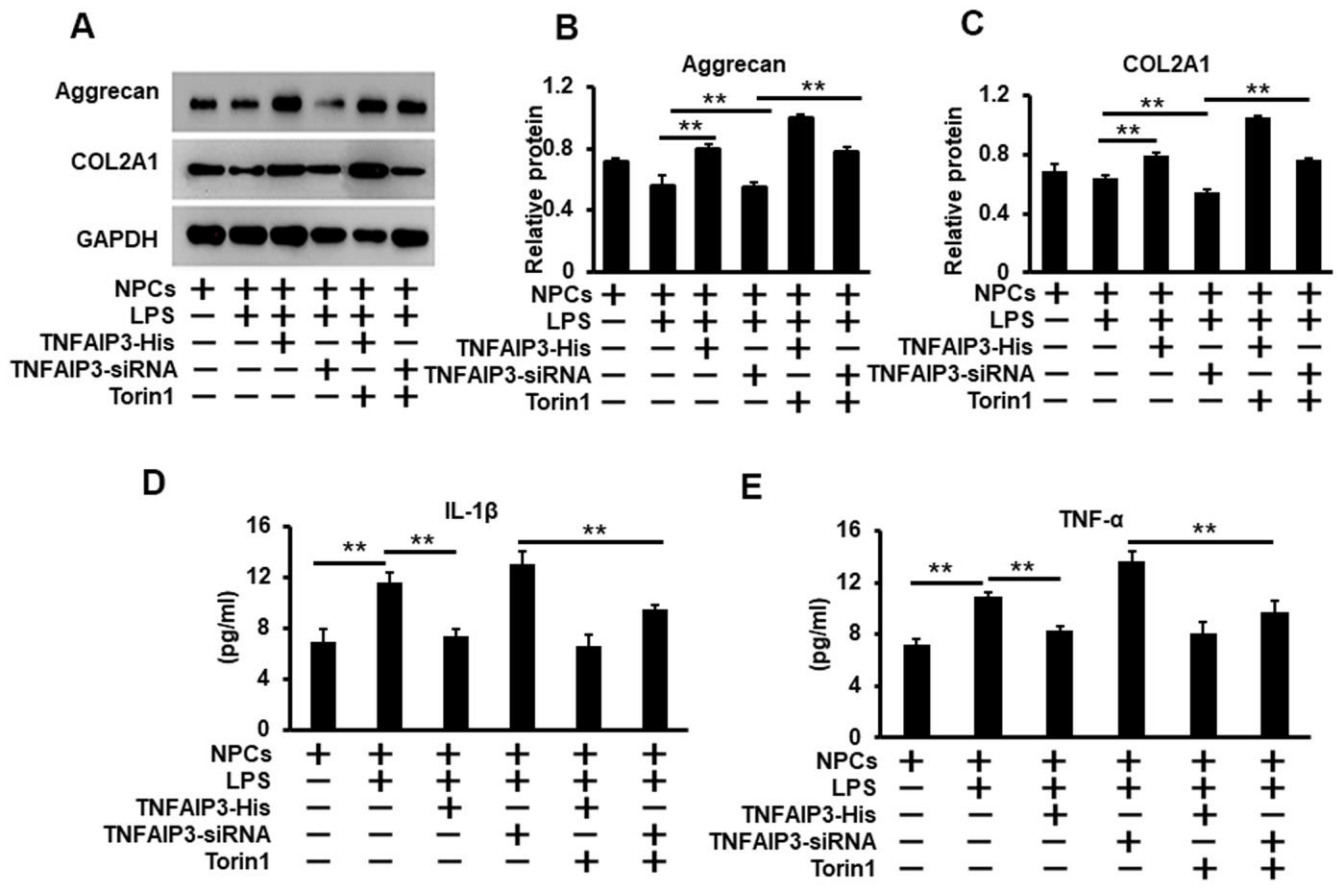
**TNFAIP3 inhibits the inflammatory response and promotes the synthesis of the extracellular matrix.** (**A**, **C**) TNFAIP3 enhanced matrix production by the inhibition of mTOR signaling. Matrix production was measured by Western blot for the levels of COL2A1 and Aggrecan (A) With their quantification normalized to GAPDH (**B**, **C**). (**D**, **E**) TNFAIP3 decreased the protein level of inflammatory cytokines IL-1β (**D**) and TNF-α (**E**) in the supernatants of the NPCs culture as quantified by ELISA. Data are represented by the mean ± standard deviation of 3 independent replicates. *** P<0.01, ** P<0.05, * P>0.05 by the Student t-test. Torin1 treated cells at 20 nM for 24 h.

To further evaluate the function of TNFAIP3 on the inflammatory response and extracellular matrix production in human NPCs and its underpinning mechanism. We continued to test inflammatory cytokines (IL-1β and TNF-α) production and extracellular matrix of aggrecan and type II collagen under the condition of mimicking IVDD. As we previously established the LPS-stimulated IVDD model in human primary NPCs, LPS reduced matrix protein production of aggrecan and type II collagen as previously reported (Aging 2019, 11:7294). We found that over-expression and knockdown of TNFAIP3 respectively increased and decreased the expression of both aggrecan and type II collagen (Figure 6A–6C), indicating TNFAIP3 maintains the NPCs function. Furthermore, inhibition of mTOR signaling further increased the production of both aggrecan and type II collagen. In addition, the ELISA assay was used to test the pro-inflammatory cytokines such as IL-1β and TNFα. The results showed that LPS treatment induced the expression of both factors. However, TNFAIP3 reduced the expression of both factors under inflammatory conditions by LPS induction. Moreover, inhibition of mTOR signaling reversed TNFAIP3 inhibition on both IL-1β and TNF-α production (Figure 6D, 6E). These data indicated that TNFAIP3 diminished the levels of inflammatory cytokines and enhanced the synthesis of extracellular matrix in inflammatory human NPCs.

## DISCUSSION

The results of this study showed that TNFAIP3 significantly reduces inflammatory response and ameliorates the degradation of the extracellular matrix by promoting autophagy in LPS-induced-inflammatory human NPCs that mimics IVDD. Further, pharmacological inhibition of mTOR signaling can significantly reverse the effects of TNFAIP3 knockdown, including inhibition of autophagy and extracellular matrix production, as well as the promotion of inflammatory response. These findings revealed the relationship between degeneration process, inflammatory response, and autophagy in human NPCs, in which TNFAIP3 inhibits the mTOR signaling to ameliorate IVDD.

Previous studies have confirmed that many factors such as oxidative stress, trauma, and smoking cause the degeneration of intervertebral disc by inducing pro-inflammatory cytokines before the degeneration process [3–7]. Ding F et al. found that pro-inflammatory cytokines inhibited the synthesis of the extracellular matrix, leading to the degeneration of human NPCs [18, 19]. Therefore, it is necessary to study mechanisms of inflammation on disc degeneration. It has been reported that inflammatory cytokines induced the production of ROS in a variety of cell types, resulting in tissue damage [20]. Simultaneously, autophagy as an immune defense system inherent in the body prevents the generation of ROS stimulated by inflammation in innate immunity [21, 22], indicating that autophagy may play an important role in inflammation. The relationship between pro-inflammatory cytokines and autophagy in disc cells is new research. Jansen HJ et al. found that autophagy increased in adipose tissue of obese individuals, regulating pro-inflammatory cytokines expression [23]. Delk NA et al. found that IL-6 induces autophagy in bone metastatic prostate cancer cells [24]. Other studies have also shown that autophagy-related genes in rat annulus cells pretreated with IL-1β and TNF-α were significantly increased [9]. However, a recent study by Jiang et al. showed that the ratio of LC3-II to LC3-I decreased in degenerative NPCs [14]. Similarly, our results also indicated that the ratio of LC3-II to LC3-I expression in LPS-induced human NPCs was lower than the control group after treatment for 24 hours. The difference in LPS-induced autophagy has aroused our great interest. We speculated that the expression of autophagy during LPS stimulation may depend on the time of LPS induction. Ji QJ et al. has reported that at a specific concentration stimulated by LPS, the level of autophagy first increased and then decreased in lung epithelial cells for 12-16 hours [25]. Chen C et al. has also found that LC3II protein expression in the liver increased significantly compared with baseline, which peaked at 6 h and then decreased [26]. These results indicate that autophagy plays an important role in the early inflammation responses, and decreased autophagy may be a valuable factor resulted in the degeneration of disc cells with chronic inflammatory stimulation. Therefore, up-regulating autophagy may be a potential strategy for improving severely degenerative intervertebral disc or age-related degeneration. In addition, the effect of autophagy on human NPCs has been controversial. Jiang L et al. found glucosamine protected the degeneration of NPCs by inducing autophagy and further demonstrated that glucosamine activates autophagy in a dose-dependent manner by inhibiting the phosphorylation of mTOR [27]. However, under the condition of serum deprivation, excessive autophagy is downregulated by the production of ROS in NPCs, improving the survival of NPCs, while excessive autophagy triggers the apoptosis of NPCs [28]. These results showed that different levels of autophagy were involved in NPCs. In this study, we found that autophagy decreased in LPS induced human NPCs, leading to the up-regulation of inflammatory factors and the degradation of the extracellular matrix. However, these effects can be reversed by promoting autophagy after TNFAIP3-His treatment in LPS stimulated human NPCs. Therefore, an appropriate level of autophagy is necessary for the survival of NPCs under different stimulation conditions, which may delay the degeneration of inter-vertebral disc.

As we all know, autophagy called “self-eating” is important for maintaining the homeostasis and integrity of cells [29, 30]. In this research, we found that the degeneration of human NPCs enhanced in LPS-induction. Previous studies have shown that oxidative stress caused by inflammatory stimulation promoted the apoptosis of NPCs during disc degeneration. Wang D et al. found the level of proteoglycan in NPCs treated with IL-1β decreased, which triggered apoptosis [31]. Yang D et al. also found that in H_2_O_2_-treated NPCs, the mRNA levels of aggrecan and type II collagen decreased and apoptosis increased [32]. Interestingly, Wei Y et al. found that promoting autophagy can inhibit the apoptosis of human NPCs [33]. It is worth noting that autophagy and apoptosis are involved in IVDD. We speculated that increased autophagy can protect human NPCs from apoptosis, thereby delaying the degeneration of human NPCs. Therefore, maintaining the relationship between autophagy and apoptosis may be an effective method for treating disc degeneration. The results in this study proved that the degradation of the extracellular matrix decreased in TNFAIP3-His treated NPCs by promoting autophagy. Nevertheless, it is unclear whether TNFAIP3 simultaneously regulates autophagy and apoptosis in human NPCs, and it is worthy of further research.

As a key negative regulator of immune cell function and homeostasis, TNFAIP3 plays a vital role in inflammation response. In addition, TNFAIP3 protects cells from death in an ill-defined manner. Jessica Vetter et al. found that the ubiquitin-editing enzyme TNFAIP3 can control the live and death of natural killer (NK) cells through the regulation of mTOR activity [34]. In this study, we found TNFAIP3 up-regulated in degenerative disc tissues, reversing the degeneration of disc by promoting autophagy. Yu Matsuzawa et al. also found that TNFAIP3 promoted the survival of CD4 T cells by restricting mTOR and promoting autophagy [35]. In addition, we also found TNFAIP3 up-regulation in LPS-induced human NPCs, but the expression of inflammatory cytokines was higher than that of the control group. We speculated that TNFAIP3 is rapidly up-regulated in the early stage of LPS-induced and then decreased, which is not enough to prevent the subsequent inflammatory response. Liu et al. has found that the expression of TNFAIP3 peaked at 4 to 7 hours after endotoxin injection, and declined after 10 hours. It was severe at 0.5-4 h for the pathological damage of liver tissue and was less than before at 7 h after the injection of endotoxin, but it was still more serious than the normal control group. These results showed that TNFAIP3 induced by LPS is insufficient for persistently inflammatory stimulation, and TNFAIP3 may be important for the metabolism in nucleus pulposus cells, providing a new research idea for the treatment and prevention of degenerative diseases.

Further study on TNFAIP3 in human NPCs aims to understand the mechanism by which TNFAIP3 blocking these pathways and elucidates the specific domain where TNFAIP3 binds to signaling molecules in human NPCs. In conclusion, our findings initially reveal the relationship between TNFAIP3, autophagy, inflammation and disc degeneration, and further illuminate the mechanism by which TNFAIP3 regulates autophagy of human NPCs in inflammatory stimulation. In summary, our results reveal a new function of TNFAIP3, which may provide a potential and an attractive therapeutic strategy for degenerative diseases.

## MATERIALS AND METHODS

### Cell collection and culture

The collection and use of human NP specimens have been approved by the Ethics Committee of Chongqing Medical University before surgery. All patients signed written informed consent. All samples were obtained after surgery at the First Affiliated Hospital of Chongqing Medical University. The control group was obtained from patients with lumbar vertebral fracture (LVF) (n>5), and the degenerative group was obtained from patients with intervertebral disc degeneration (IVDD) (n>15). Pfirrmann classification was used to define degeneration grades of IVDD by MRI scans before surgery [36]. Pfirrmann grade I or II was defined as a relatively normal discs in patients with LVF, and Grades III-IV was defined as degenerative disc cells in patients with IVDD.

The NPCs isolated from patients' inter-vertebral disc tissue after surgery was quickly digested with 0.25% trypsin solution at 37° C for 30 mins, then digested with 0.2% type II collagenase for 4 hours. Tissue debris was removed with a 200 μm filter after digested. The mixture was centrifuged at 1,000 × g for 5 min, and then the cells were cultured with DMEM/F 12 medium (Gibco, USA) containing 10% FBS (Corning, USA) and 1% penicillin-streptomycin at 37° C, 5% CO_2_. The 2^nd^ passaged NPCs were used for following experiments.

### Cell treatment and transfection

The human NPCs were cultured with DMEM/F-12 medium with 10% FBS and transfected Ad-TNFAIP3 which was constructed with pHB Ad-EF1-MCS-GFP system for 24 h, and then were treated with 200 ng/ml LPS (Sigma, USA), simultaneously stimulated with Torin1 (20 nM) for 24 h. Over-expression TNFAIP3 (TNFAIP3-His), knockdown TNFAIP3 (TNFAIP3-siRNA) and Adenovirus vector without TNFAIP3, (Negative control, NC) were designed and purchased from HANBIO (Shanghai). Cells were transfected according to the manufacturer's instructions.

### Immunohistochemistry (IHC)

The human NP tissues collected from patients with lumbar vertebral fracture (LVF) and IVDD were fixed in 4% paraformaldehyde at 4° C for 48 h, and then were embedded in paraffin. According to the manufacturer's protocol, the IHC was performed by immunohistochemical kit (Wuhan Boster Biological Technology, Ltd., Wuhan, China), Briefly, the tissues samples were baked and dewaxed. Then antigen was retrieved. Next, the tissues were incubated with rabbit primary anti-TNFAIP3 (EPR2663, Abcam) at 4° C overnight. Then, the tissues were incubated with the corresponding secondary antibody IgG-HRP (155000) for 60 mins. DAB staining was added under the microscope and counterstained with hematoxylin for 2 mins. The sample was sealed with a neutral resin for 2 d. The images were collected with a microscope (Leica DM4 M, GERMARY).

### The observation of autophagosomes by TEM in NPCs

The transmission electron microscopy (TEM) was used to observe the autophagosomes in NPCs. The samples were fixed in 4% glutaraldehyde after centrifugation twice, then cut into thin sections (60-80 nm), mounted on a copper grid, and post-stained with uranyl acetate and lead citrate. Images were captured using the JEOL JEM-1400PLUS.

### The detection of autophagy activity by GFP-LC3 assay

The GFP-LC3-adenovirus was used to detect the autophagy activity in NPCs. After transfected for 24 h, the levels of autophagy were evaluated with green fluorescent puncta of autophagosomes through laser confocal microscopy.

### The detection of inflammatory cytokines by ELISA

The ELISA assay was used to detect inflammatory cytokine in NPCs. Samples supernatants were collected from each group of cells after treated for 48 h. The samples supernatants were centrifuged at 1,200 × g for 5 min to remove cell debris and stored at -80° C until use. The levels of TNF-α and IL-1β in cultured supernatants of human NPCs was precisely measured with human ELISA kits according to the manufacturer's instructions, respectively. The optical density was determined with a BIO-RAD Microplate reader (CA, USA), and the absorption was measured at 450 nm. A standard curve for each measurement was established using a cytokine standard provided by the ELISA Kit. Each experiment was performed three times independently.

### The detection of protein level by Western blot

Cell protein was dissociated using RIPA lysis buffer, and then cell protein concentration was measured by BCA Protein Assay Kit (Solarbio, PC0020). The proteins (35-40 μg) were separated using Nu PAGE 6-12% Bis-Tris-SDS gel (Invitrogen) and transferred to PVDF and NC membrane (0.45, 0.22 mm). Membranes were blocked with 4% Nonfat-Dried Milk for 2 h at room temperature and incubated with indicated primary antibodies anti-TNFAIP3 (EPR2663, Abcam), anti-COL2A1 (ab 34712, Abcam), anti-Aggrecan (ab 3778, Abcam), anti-LC3A/B (CST, D3U4C), anti-TNFAIP3 (ab 92324, Abcam), anti-phospho-mTOR (S 2448) (AP0094, Abclonal), anti-mTOR (A 2445, Abclonal), at 4° C overnight. All antibodies were used according to the manufacturer's recommendations. After washing the membranes three times with Tris-buffered saline containing 0.1% Tween 20 (TBST), the membranes were incubated in the appropriate secondary antibody (1:5,000 dilution) (Jackson Research, PA) for 2 h at room temperature, and then washed 3 times with PBS for 5 minutes each time. Finally, the membranes were measured by an ECL plus reagent (Solarbio, PE0010) and the results were quantified using ImageJ software (National Institutes of Health).

### The detection of mRNA level by RT-qPCR

Collected the cells from Con group, LPS group, LPS+NC group, LPS+TNFAIP3-His group, LPS+TNFAIP3-siRNA group, the mRNAs of human NPCs were isolated using TRlzol Reagent (Invitrogen, USA) according to the manufacturer's instructions. Genes expression was determined by real-time PCR using Prime-Script® RT reagent kit (Takara, DRR037A) and SYBR® Green Real-Time PCR Master Mix (TOYOBO, QPK-201). The relative expression of the gene from the control was evaluated by the 2-ΔΔCT method. The Primers for target gene were as follow: (Table 1).

**Table 1:** Primer pairs for qPCR.

**Gene**	**Primer sequence**
TNFAIP3	F5' AGAGCAACTGAGATCGAGCCA 3'
	R5' CTGGTTGGGATGCTGACACTC 3'
GAPDH	F5' GCACCGTCAAGGCTGAGAAC 3'
	R5' TGGTGAAGACGCCAGTGGA 3'

All the primers were synthesized by Takara (Takara, China).

### Statistical analysis

All experiments were performed in three independent replicates, and the results were shown by the mean ±SD. Statistical analysis was performed by t-test using software SPSS 19.0. P-value was defined as significant, *** indicates p < 0.01, ** indicates p < 0.05, * indicates p > 0.05. Error bars indicate ±SD.
